# Protective Factors against Suicide and Violence against Women in Azerbaijan: Shedding light on Suicide in Muslim-Majority Countries

**DOI:** 10.1192/j.eurpsy.2023.2356

**Published:** 2023-07-19

**Authors:** D. Alonzo, P. Zubaroglu-Ioannides

**Affiliations:** 1 Fordham University, West Harrison, United States

## Abstract

**Introduction:**

Suicide rates in Azerbaijan rank among the top 3 highest of all Muslim majority countries. Further, approximately 40% of women in Azerbaijan report being physically or sexually abused. Women experiencing interpersonal violence report higher rates of suicide ideation and attempts (34%) than women in the general population. No prior studies have specifically explored protective factors against suicide and interpersonal violence in Azerbaijan.

**Objectives:**

This study aims to address this gap and to identify culturally relevant protective factors against suicide and violence against women in Azerbaijan.

**Methods:**

A total of 51 women with lived experience and mental health providers participated in either in-depth qualitative interviews or focus groups. The interview guide for the focus groups and one-on-one interviews were developed by the study PI. A list of questions served as the basis for the discussions and was revised and expanded as the groups progressed. For the qualitative analyses, conventional content analysis following a systematic process of coding and classification was utilized.

**Results:**

Three main protective factors against suicide were identified: 1) psychological support (33%); 2) psychoeducation to raise awareness of suicide and reduce stigma (28%); and 3) providing financial opportunities/supports (10%) and for violence against women 1) advocacy (28%); 2) psychological support (28%); and 3) changing cultural values (17%).

**Conclusions:**

This study fills a much-needed gap in our understanding of suicide and violence against women in Muslim populations. Our findings suggest that for intervention to be relevant and effective, prevention programming needs to span micro, mezzo, and macro levels.

**Disclosure of Interest:**

None Declared

